# Can Biochar and Phytoextractors Be Jointly Used for Cadmium Remediation?

**DOI:** 10.1371/journal.pone.0095218

**Published:** 2014-04-16

**Authors:** Huanping Lu, Zhian Li, Shenglei Fu, Ana Méndez, Gabriel Gascó, Jorge Paz-Ferreiro

**Affiliations:** 1 Key Laboratory of Vegetation Restoration and Management of Degraded Ecosystems, South China Botanical Garden, Chinese Academy of Sciences, Guangzhou, China; 2 University of Chinese Academy of Sciences, Beijing, China; 3 Departamento de Ingeniería de Materiales, Universidad Politécnica de Madrid, Madrid, Spain; 4 Departamento de Edafología, Universidad Politécnica de Madrid, Madrid, Spain; Massey University, New Zealand

## Abstract

Phytoremediation of soils contaminated with cadmium was tested after liming (CaO) or biochar addition, using red amaranth (*Amaranthus tricolor* L.) as test plant species. Two biochars with contrasting characteristics were prepared from two feedstocks and added to the soil at a rate of 3% (w:w): Eucalyptus pyrolysed at 600°C (EB) and poultry litter at 400°C (PLB). Liming was carried out in two treatments (CaO1) and (CaO2) to the same pH as the treatments EB and PLB respectively. Total plant mass increased in soils amended with PLB and with a mixture of PLB and EB; however this was not sufficient to increase the efficiency of phytoextraction. Bioavailable and mobile fractions of Cd diminished after liming or biochar addition. Our study infers that, both the amount of Cd immobilized and the main mechanism responsible for this immobilization varies according to biochar properties.

## Introduction

Industrialisation and technical advances have led to an increase in the use of heavy metals. Anthropogenic activities, including smelting, mining, use of pesticides, fertilisers and sludge are responsible for the input of high levels of heavy metals into the soil. In the last years there is an increasing concern about soil heavy metal pollution. Soil heavy metal pollution poses a risk to the environment and to human health due to biomagnification (increases in metal concentration as the element passes from lower to higher trophic levels). An additional concern is that, contrary to organic substances, heavy metals are non-degradable and accumulate in the environment.

In particular, cadmium pollution is a concern in vast areas of China and some areas in Japan, Taiwan and other countries, rendering valuable farmland into non-cultivated areas. Cadmium is known to originate from lithogenic sources in areas with high abundance in zinc and lead ores. However, in the last years the agricultural use of fertilisers and the addition of sewage sludges to the soil have resulted in an increase in soil Cd concentrations. In general plants tolerate Cd better than human and animals and Cd toxicity in plants appears at higher concentrations than in animals or humans. In humans, excessive Cd intake can cause the “Itai-itai disease” and has also been related to some types of cancer or to kidney damage.

In the last years there has been an increasing interest in the use of biochar to tackle soil heavy metal pollution [1], [2], [3]. Biochar influences a number of biogeochemical processes and in general there has been reported a positive effect on plant productivity [4] while simultaneously mitigating greenhouse emissions (as CO_2_ and N_2_O) from soil. Several co-benefits may result from the use of biochar including positive effects on soil field capacity, nutrient availability, fertilizer use efficiency, pH (“liming effect”), cation exchange capacity (CEC) and soil biological properties [5]. Therefore, in the past few years, there has been a growing interest in the use of biochar as a soil amendment. The potential of biochar to immobilize toxic substances, in particular cadmium, from different types of soil is well documented [2], [3], however, more uncertainties prevail about the mechanisms involved in heavy metal immobilization and their relative importance.

Biochars using plant materials as feedstocks are considered a soil conditioner, due to their low leachable nutrient contents [6]. On the contrary, manure-derived biochar can act as soil fertilizer and conditioner due to NPK release [7].

On the other hand, phytoextraction consists in the use of hyperaccumulator plants to remove heavy metals from the environment. This is a relatively new technology developed from 1990 onwards. Because of its low costs, phytoremediation can be considered as a relatively attractive technique to restore or partially decontaminate a site compared to other options, e.g. [8], [9]. Other advantages of this technique would include its good perception among the general public. Plant species used for phytoextraction must not only accumulate high amounts of the target element but also have a high growth rate, tolerate toxic effects of heavy metals, be adapted to local soil and climate, be resistant to pathogen and pests, be easy to cultivate and repulse herbivores to avoid food chain contamination [9], [10]. Management of soil pH (liming) and soil nutrients have been utilised in the past in order to increase metal hyperaccumulation. In particular, liming is more effective when heavy metal concentrations in the soil are too elevated to reduce plant stress [11], [12] [13]. Organic matter amendments from several sources have been jointly used with phytoremediators and results showed that organic matter had either null or positive effect in enhancing phytoremediation [12], [14].

Of the more than 400 plants identified as phytoextractors most are Ni-hyperaccumulators and only a handful of plants accumulating Cd have been identified. Moreover, most Cd-accumulating plants present slow growth rates. Recently *Amaranthus tricolor* L. has been identified as a Cd-accumulator [15]. This species presents the main advantage of a rapid growth and a relative high yield compared to other Cd accumulating plants [15].

Simultaneous use of biochar and phytoremediation may seem contradictory, because until now most research results indicate that biochar reduce the bioavailability of heavy metals [2], [3] but plants need high concentrations of soluble metals to extract and to accumulate them. In fact, exploration of the efficiency in the joint use of biochar amendments and phytoremediation, has been attempted before [16], [17] for the case of Cd using *Brassica napus* L. as phytoextractor and Miscanthus biochar [16] or for the case of multicontaminated soils using different biochars and plant species [17].

Based on the above considerations we performed an experiment with the following aims: a) To explore the possibilities of biochars with contrasting characteristics on Cd remediation b) To explore the possibility of combining biochar with phytoremediation c) To deepen our mechanistic knowledge about how biochar contributes to heavy metal immobilization, using lime controls.

## Material and Methods

### Ethics statement

No specific permissions were required for collecting the soil, poultry litter or Eucalyptus used in this study.

Our study did not involve the use of endangered or protected species.

### Soil sampling and characteristics

The soil was collected from the surface layer (0–20 cm) of a cropland area, near a waste landfill site in the suburb of Guangzhou, China (23° 07′ N and 113° 21′ E). Guangzhou is located in the subtropical humid area having an average annual temperature of 12.7°C and annual average precipitation of 1700 mm. According to FAO, the soil is classified as a fimic anthrosol.

Part of the soil was sieved to 2 mm to conduct general analyses, while the rest was sieved to 10 mm to conduct the experiment. Before the starting of the pot experiment, the soil had a total organic carbon content of 1.98%, total nitrogen content of 0.142%, pH of 6.00, total phosphorus of 690 mg kg^−1^, available phosphorus of 126 mg kg^−1^ and total Cd 6.1 mg kg^−1^.

### Preparation and characterization of biochar

Two biochars were used in this experiment using poultry litter and eucalyptus as feedstocks. Poultry litter was collected at the Experimental Poultry Farm of South China Agricultural University (23° 09′ N and 113° 21′ E) located at Guangzhou, China. Chicken at this facility are organically bred and fed with a mixture of corn, wheat bran and soybean pulp. After collection, litter was dried in an oven (70°C) for 24 hours and sieved to 1 mm.

Eucalyptus (*Eucalyptus urophylla* S.T. Blake) wood was collected at Heshan Hilly Land Experiment Station of the Chinese Academy of Sciences in Heshan, China (22° 84′ N and 112° 54′ E). Wood was collected from branches of four different individuals, mixed together and brought to the South China Botanical Garden where it was ground to wood chips with a particle size smaller than 0.5 mm.

Biochars were prepared as described by [18] with samples being pyrolysed by increasing the temperature to 400°C (poultry litter biochar) or 600°C (Eucalyptus biochar) at a rate of 10°C min^−1^. The final temperature was maintained for 2 h. Different temperatures were used in the preparation of the biochars as our main aim was to produce two biochars with contrasting characteristics.

### Mesocosm experiments

On the 13^th^ April of 2013, a mesocosm using a fully replicated randomised experiment was set up in a greenhouse in South China Botanical Garden. Each mesocosm consisted in a pot filled with 1.5 kg of soils in pots with plants and 500 g of soil in pots without crops. The reason for using different amounts of soil was to minimise the amount of biochar prepared, while allowing the crops to have enough soil to grow. The soils were watered to 60% of field capacity and watered daily to account for moisture losses.

The experiment studied two factors, namely the type of amendment and the presence/absence of phytoremediator, having 4 replications per treatment. The treatments in the amendment factor were: Control (C), poultry litter biochar (3% w/w) (PLB), eucalyptus biochar (3% w/w) (EB), poultry litter biochar (1.5% w/w) + eucalyptus biochar (1.5% w/w) (BB), liming with CaO as necessary to increase soil pH as much as EB (CaO1) and liming with CaO as necessary to increase soil pH as much as PLB (CaO2).

With respect to the factor involving presence/absence of phytoremediator, half of the mesocosm were planted with 3 seeds of red amaranth (Amaranth tricolor L.), while in the remainder half the vegetative cover was absent.

After 60 days soils and plant were collected for analyses.

### Soil analysis

Soil pH was determined using a 1∶2.5 soil:water ratio. Total carbon and total nitrogen were determined using using a vario ISOTOPE CUBE elemental analyzer (elementar, Germany). Plant-available metals were extracted from treated soil using diethylenetriaminepentaacetic acid–CaCl_2_–triethanolamine (DTPA) as described elsewhere [19]. The mobile forms of heavy metals were extracted using 0.1 M CaCl_2_ [20].

### Plant analysis

The crops were harvested and separated into roots, stems and leaves. Then they were rinsed firstly with tap water followed by distilled water. Samples were oven-dried for 72 h at 65°C. Once dried, plant biomass was weighted and ground. For digestion, a microwave closed system (Multiwave3000, Anton Paar, Austria) was used. Samples (0.1 g) were digested with 5 mL of nitric acid and 1 mL of hydrogen peroxide in a microwave digestion system for 30 min and diluted with deionized water to 30 mL. Cadmium concentrations were analyzed using an ICP-MS spectrometer (Agilent 7700x, USA).

### Biochar analysis

pH and TOC in the biochar were determined as described for the soil. Biochar nitrogen adsorption analysis to determine BET surface was carried out at 77 K in a Micromeritics Tristar 3000 (Instituto de Catálisis y Petroquímica, CSIC, Spain).

Total pore volume (V) was estimated from the amount of nitrogen adsorbed at a relative pressure. Average pore size (D) was estimated from V and BET surface using the following equation and assuming a cylindrical pore shape:

D = 4V/S_BET_.

Proximate analysis was calculated by thermogravimetry using a Labsys Setaram equipment. Samples were heated up to 600°C under an N_2_ atmosphere at a flux of 40 mL min^−1^ using a heat rate of 20°C min^−1^. Moisture content (H) was determined as weight loss at 120°C and Volatile matter (VM) was determined as the weight loss from 120°C to 600°C. At 600°C, air flux was introduced until a constant weight was reached and ashes were determined as the final weight of the samples. Fixed carbon (FC) was calculated by difference.

Cadmium in the biochars was determined as described for the soil samples.

### Phytoextraction indices

The amount of Cd transported from soil to shoots was calculated using the bioconcentration factor (BCF), [21].

Accumulation Factor  = Cd concentration plant tissue/Cd concentration in soil.

The ability of each species to translocate Cd from the roots to the shoots was calculated by the translocation index (TI) [22].

TI (%) = Cd concentration in aerial parts/Cd concentration in roots x 100.

### Statistical analysis

Statistical analyses (calculation of means and standard deviations, differences between treatments) were performed using SPSS 15.0 package. Differences of means were tested using a two-way ANOVA with the presence/absence of plant and the type of amendment (eucalyptus biochar, poultry litter biochar, CaO1, CaO2, mixture of biochar or none) as factors. Means were considered to be different when P<0.05 using the Tukey's test.

## Results and Discussion

### Biochar properties

The physical characteristics of both biochars were contrasted (Table 1). Eucalyptus biochar (EB) had a larger surface area and pore volume compared to the poultry litter biochar (PLB) due to its higher content in carbon. Biochar pore volume has been directed related before to surface area [23] and both, pore volume and surface area, are expected to have larger values in biochars prepared at higher temperatures [24]. Rouquerol et al [25] defined the internal diameter range of pores for porous solids, suggesting <2.0 nm as micropores, 2.0–50.0 nm as mesopores, and >50.0 nm as macropores. Our results showed clear evidence that the PLB was dominated by mesopores, while EB was dominated by micropores.

**Table 1:** General characteristics of the biochars used in the experiment.

	pH	Carbon (%)	Nitrogen (%)	Cd (mg kg^−1^)	Surface area (m^2^ g^−1^)	Average pore width (nm)	Pore volume (cm^3^ g^−1^)	Ash (%)	VM (%)	FC (%)
PLB	10.02	16.77	1.37	n.a.	7.418	15.406	0.0286	74.95	7.66	17.39
EB	10.40	81.03	1.07	n.a.	334.560	1.928	0.1612	1.74	2.19	96.06

VM and FC stand for volatile matter and fixed carbon, respectively.

Biochar pHs are very basic according to other works that attributed this fact to the polymerisation/condensation reactions and acidic surface group releases during pyrolysis [26]. The total Cd content of biochars was negligible. Finally, the PLB presented a higher content in ash, VM and a lower content in FC than EB due to the different composition of the raw materials and pyrolysis conditions.

### Soil properties

The addition of lime or biochar clearly modified soil pH (see Table 2). All treatments increased significantly the pH compared to the control (P<0.001). While the control soil had a pH of 6.03, this increased to 7.62 for the treatment PLB, 7.01 for EB and 7.45 for BB. The pH of PLB was significantly higher than that of EB.

**Table 2:** General properties of the soils at the end of the experiment.

	pH	Carbon (%)	Nitrogen (%)
Control	6.03±0.03 a	1.76±0.03 a	0.123±0.005 a
PLB	7.62±0.03 d	2.35±0.12 a	0.171±0.006 b
EB	7.01±0.02 b	5.19±0.52 c	0.176±0.010 b
CaO1	6.81±0.06 b	1.79±0.23 a	0.128±0.012 a
CaO2	7.08±0.12 bc	1.73±0.12 a	0.122±0.009 a
BB	7.45±0.22 cd	3.80±0.40 b	0.172±0.020 b

Different letters in the same column indicate statistical significant differences (P<0.05).

Total carbon and total nitrogen increased in BB and EB compared to the control. However, in the case of PLB only an increase in total nitrogen with respect to the control was found.

DTPA extracted-Cd and CaCl_2_ extracted-Cd can be considered respectively as the bioavailable and mobile forms of Cd present in soil. Our study showed that both fractions were affected by the type of amendment (in both cases P<0.001); however only CaCl_2_ extracted-Cd was affected by the presence of red amaranth (P<0.010). Moreover, for CaCl_2_ extracted-Cd there was an interaction phytoremediation x treatment (P<0.010). Thus, mobile forms of Cd were higher in the EB soil with amaranth with respect to the one without amaranth. This leads us to think that, at least to some extent, plants with a high affinity for heavy metals can mobilize part of the heavy metal stabilized by biochar.

**Figure 1:**
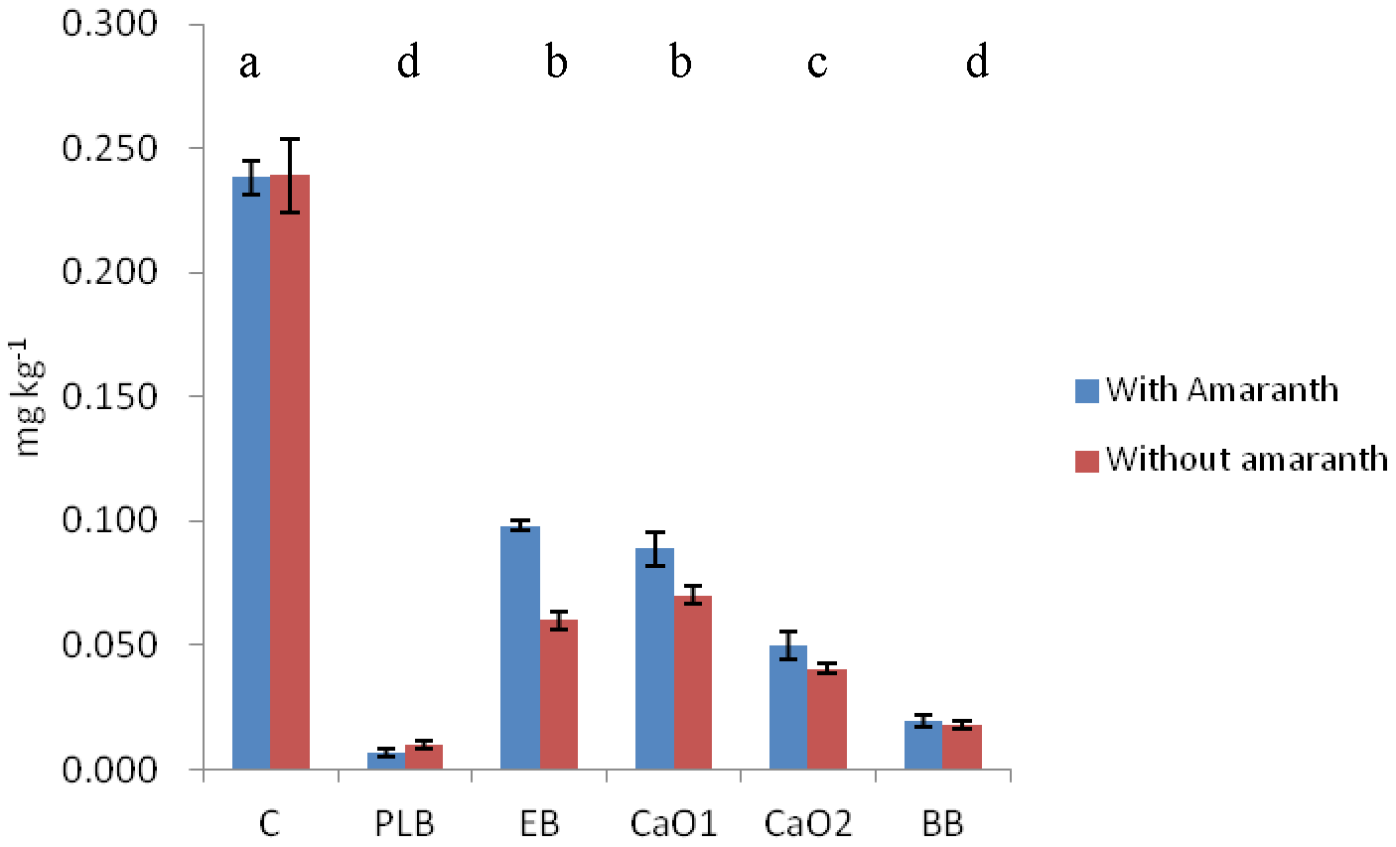
CaCl_2_ extracted Cd for all the treatments at the end of the experiment. Different letters in the same indicate statistical significant differences for the factor “type of amendment” (P<0.05).

**Figure 2:**
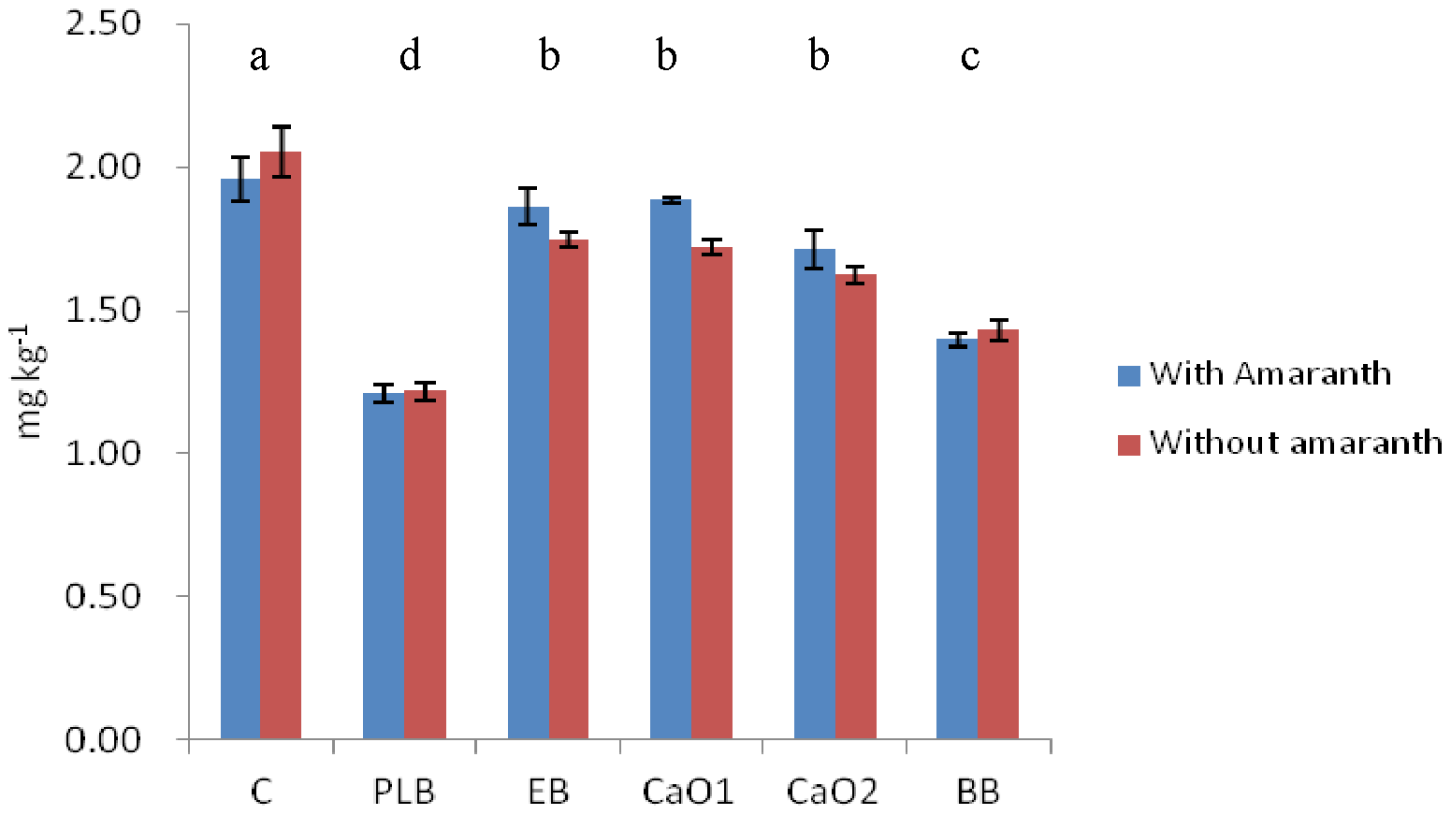
DTPA extracted Cd for all the treatments at the end of the experiment. Different letters in the same indicate statistical significant differences for the factor “type of amendment” (P<0.05).

Incorporation of biochar reduced CaCl_2_ extracted Cd compared to the control by 97, 67 and 92% respectively for PLB, EB and BB (see Figure 1). The value for PLB was similar to the one found by [16] using Miscanthus biochar. These values were 40, 10 and 29% comparing the reduction with respect to the control in the case of DTPA extracted Cd (Figure 2).

In the last years a number of studies have shown that biochars have a potential for metal adsorption and immobilization, but there is no agreement about what drives biochar effects on soil heavy metals. While some authors postulate that this effect is mainly pH mediated [27], other authors pointed out to high surface are and pore volume [23], [28], or the amount of oxygen functional groups [29]. Our study confirms the capacity of biochar to immobilize cadmium and points out to different mechanisms depending on biochar type. This experiment demonstrated that the EDTA and CaCl_2_ extractable Cd in EB was similar to that of CaO1, pointing out to Cd immobilization as caused primarily by changes in soil pH for EB and pointing to negligible effects of biochar surface area, contrary to the suggestion of other authors [30]. However, this was not the case of PLB, which had a very different EDTA and CaCl_2_ extractable Cd compared to CaO2. We would expect that Cd immobilisation in the PLB treatment was driven both by pH changes and by precipitation with mineral ash. Thus, PLB has a high content of ash, which must be enriched by mineral salts of K, Ca, Mg, C and P among other elements. Cadmium can precipitate as insoluble phosphate and carbonate salts, in particular and high pH values. Another mechanism that could explain Cd immobilization in the PLB treatment would be the presence of high oxygen-containing functional groups in the poultry litter biochar. These groups are particularly effective for heavy metal stabilization in soils with low organic carbon contents [29].

### Plant growth and Cd uptake

**Figure 3:**
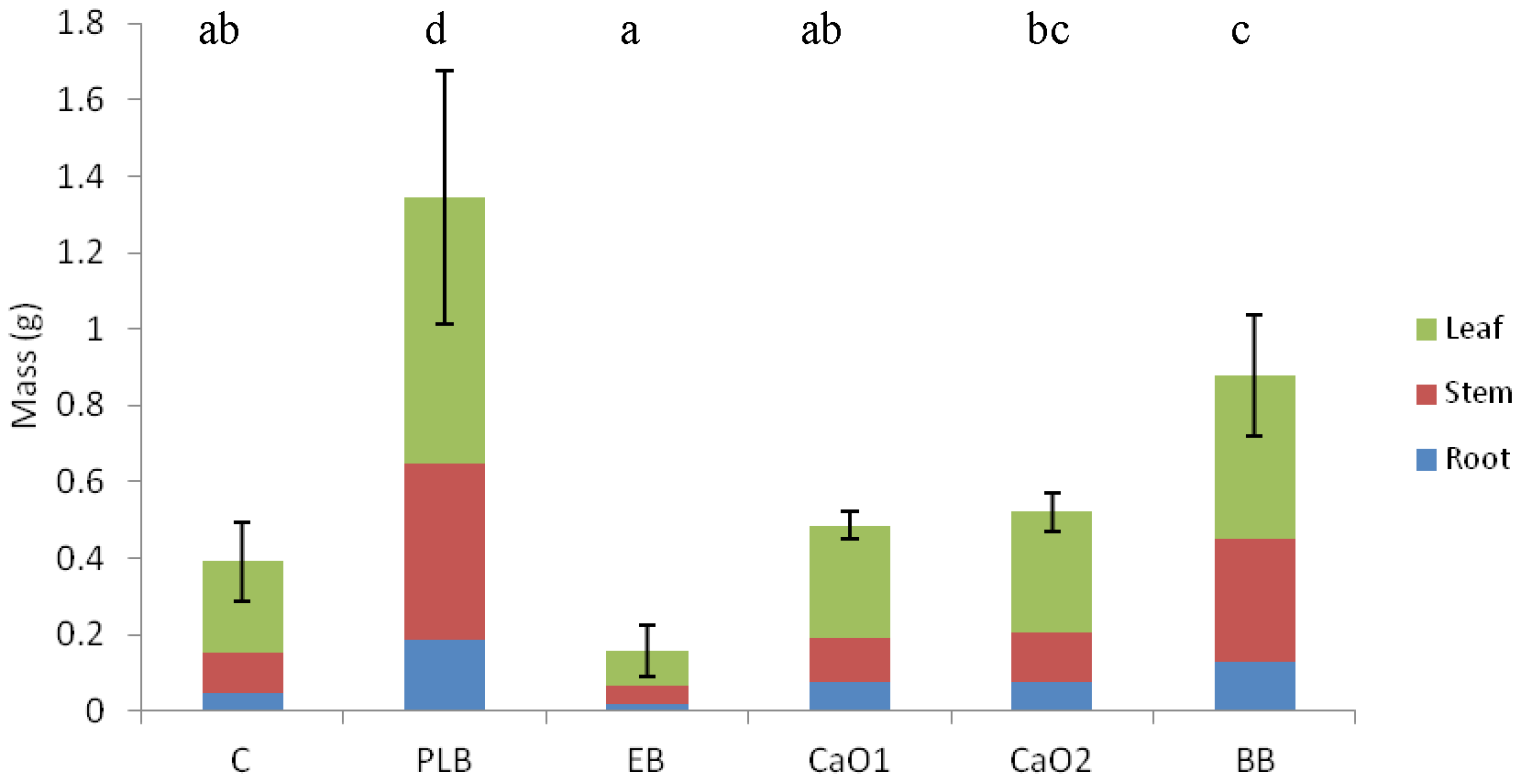
Total plant biomass at the end of the experiment. Different letters in the same indicate statistical significant differences for the factor “type of amendment” (P<0.05).

Plant biomass increased as a consequence of liming and poultry litter biochar addition (Figure 3). This way, total plant biomass was 3.44 and 2.24 times higher under PLB and BB respectively than in the control. Treatments CaO1, CaO2 and EB showed no difference with the control. We would like to highlight that plant height was higher in all treatments with respect to the control for one month following the start of the experiment (data not shown), with the addition of two different biochars (BB) performing better than any other treatment. This suggests that at least in the initial state of growing there could be some synergistic effect between different types of biochar.

Cadmium concentration in the different plant tissues was reduced by every treatment. Concentrations diminished more for PLB and for BB (with both treatments having similar values) than in EB. The concentrations of Cd in the root, shoots and leaves of the plants were in all cases over the range of concentrations (see Table 3) that is usually considered as excessive or toxic (5–30 mg kg^−1^, [31]) for humans, but we did not observe any signs of Cd toxicity in the plants.

**Table 3:** Phytoremediation parameters and Cd concentration in different plant tissues at the end of the experiment.

	BCF	TI	Root Cd concentration	Leaf Cd concentration	Stem Cd concentration
Control	12.76±1.90 a	120.27±5.42 a	76.40±4.65 a	108.57±7.87 a	52.15±7.87 a
PLB	2.17±0.42 d	70.74±20.57 b	17.79±0.58 d	15.65±1.78 d	7.31±1.78 d
EB	8.12±0.78 b	93.22±15.39 ab	53.62±4.90 b	57.77±2.47 b	30.33±2.47 b
CaO1	5.67±0.80 c	79.31±7.68 b	42.03±3.12 bc	39.27±3.12 c	17.57±3.12 c
CaO2	4.05±0.26 cd	80.21±9.89 b	29.91±1.45 cd	28.53±0.83 cd	12.07±0.83 cd
BB	2.71±0.20 d	71.80±10.27 b	22.05±1.67 d	21.64±0.73 d	7.77±0.73 d

Different letters in the same column indicate statistical significant differences (P<0.05).

### Quantification of phytoextraction efficiency

**Figure 4:**
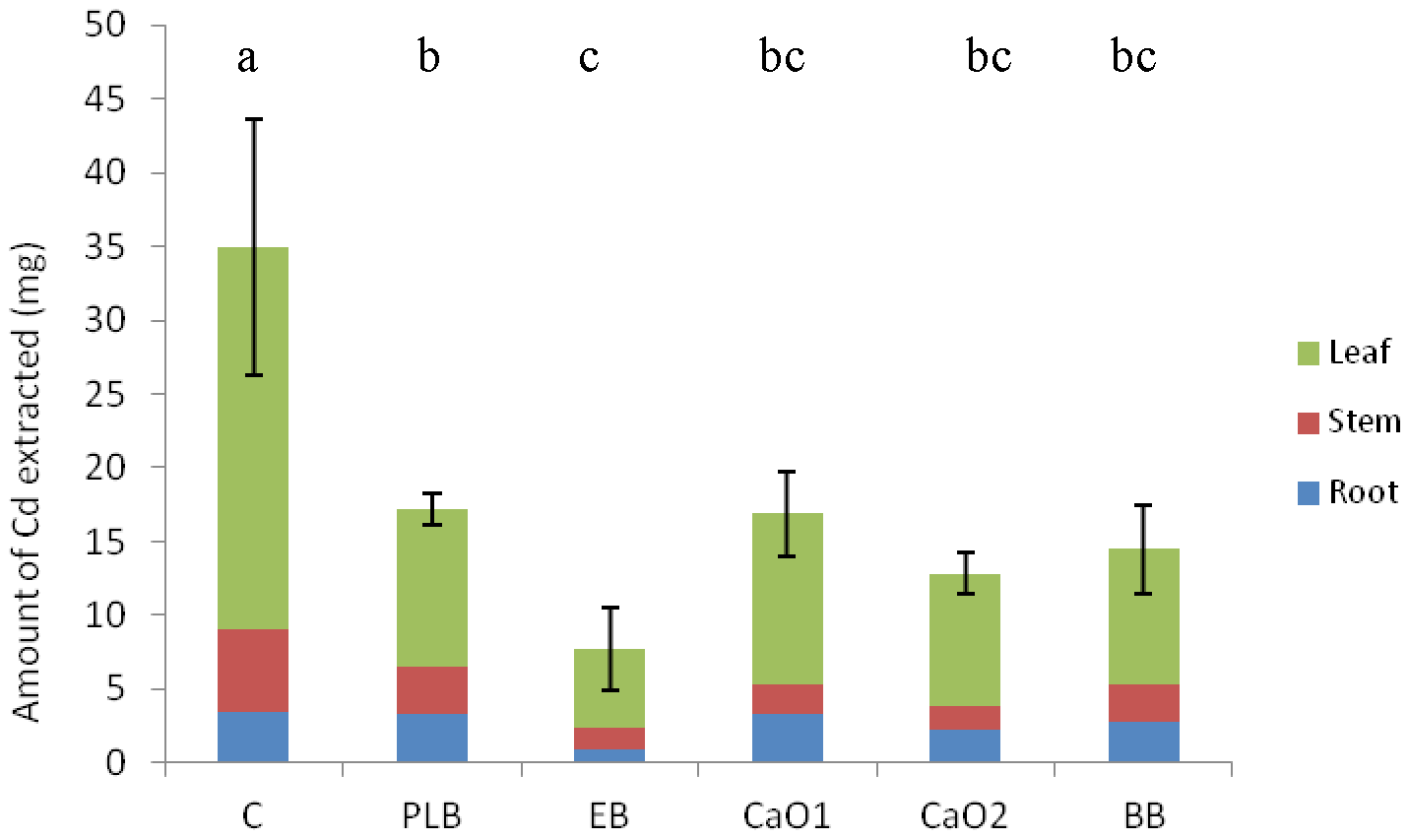
Total amount of Cd extracted by plants. Different letters in the same indicate statistical significant differences for the factor “type of amendment” (P<0.05).

All treatments extracted significantly less Cd from the soil than the control (P<0.001) as shown in Figure 4. EB treatment removed only 22% of the Cd accumulated by Amaranthus in the control, while this percentage was 49% in the case of PLB.

The translocation index (TI) was 120.5 in the control soil and dropped to 71.0 for PLB (see Table 3). The bioconcentration factor (BCF) varied from 14.76 in the control soil to 2.17 in PLB. The treatments EB, CaO1, CaO2 and BB also had a lower transfer factor than the control soil, being 8.12 5.67, 4.05 and 2.71 respectively.

The values of TI for EB and the control soil were similar probing that EB did not inhibit heavy metal transportation from roots to shoots, but did from soil to shoots (see BCF values). On the other hand, liming and PLB inhibited both, transportation from soil to roots and from roots to the aerial parts of the plant.

Both indices, TI and BCF are important in screening hyperaccumulators species. Yoon et al. [32] recommends the use of plants that have a TI of more than 100% and BCF values higher than 1. While all our treatments had BCF values higher than 1, the low values of TI would not make realistic to combine amaranth with biochar for phytoextraction purposes due to the excessive amount of time required. However, the increase of biomass observed with PLB and BB, could have some useful implications that will be next addressed.

### Significance of our study

Based on our study, it seems that using biochar alone as a soil amendment is a promising technique to immobilize Cd in polluted areas, but a careful choice of feedstock and pyrolysis temperature should be made. A study on five sites concerning rice grown in polluted areas demonstrated that biochar can reduce rice Cd uptake through a reduction in Cd mobility [1]. However, PLB would have a greater potential than the wheat straw biochar used by [1] as its use resulted in greater heavy metal immobilization, as measured by CaCl_2_ extraction.

Other experiments with Cd and biochar have shown that Cd mobility and plant availability can be reduced with increasing doses of biochar, at least up to an amendment of 10% biochar [16]. In addition to its use as a phytoremediator, amaranth has been used for as forage species or for human consumption. The values obtained in this study for Cd concentrations in the leaves of plants (>10 mg kg^−1^) would discourage the use of amaranth for this purpose, even in the treatments with lower leaf Cd concentration.

In the last years much research about biochar has been emerged and some unintended consequences have been identified including the potential presence of toxic compounds in biochars [33] and increasing difficulties for weed control due to pesticide adsoption on biochar surface [34]. Our study is, to our knowledge, pioneering (but see also [14], [17]) with respect to addressing a joint use of phytoremediation and biochar for the remediation of heavy metal polluted soils and raises awareness over a new unintended consequence (limitation on the use of phytoextractors), which can arise from biochar management. Our results point out that biochars could impede the transfer of heavy metals to phytoextractors and slow down remediation processes. However, these results should be carefully extrapolated to the field scale, as the yield metal extracted by phytoremediators can vary greatly among field and mesocosm studies [35].

Our study did not account for other potentially beneficial effects which are concomitant to the use of phytoremediation and are derived from the establishment of a plant cover. In this aspect, introduction of vegetation in a polluted area can help to prevent erosion or contaminant leaching. In addition, it could be expected that the vegetation will help the improvement of soil biological properties. Moreover, the harvested biomass of the phytoremediator could be used as feedstock for biochar production. This option would only be feasible if the feedstock biomass does not contain excessive levels of heavy metals. Indeed, Méndez et al. [2] have demonstrated that after pyrolysis of a sewage sludge containing heavy metals, the major part of the metal was immobilized in the biochar and as a consequence, addition of sewage sludge biochar did not result in increased mobile fractions of metals in soil. However, metal concentrations in a biochar prepared using a metallophyte as feedstock would presumable be higher than in the sewage sludge used by Méndez et al. [2]. Carbon sequestration is another benefit that could arise from the combined use of phytoextractors and biochar. Thus, we could devise some situations where it could be advantageous to combine phytoextraction with biochar amendment. This type of scenarios would include soils with low organic matter contents and with acidic pH values which would compromise the growth of phytoextractors.

There are two big interrogations regarding the future use of biochar and phytoremediation as joint strategies to address heavy metal pollution. Firstly, our experiment has only considered a single heavy metal (cadmium). According to other studies [3], [36] biochar has a moderate affinity for Cd which would make our results difficult to extrapolate to heavy metals that are not so likely to be immobilized by biochar or to multicontaminated soils. Secondly, the development of biochar-based fertilizers [37] could change the present paradigm of soil heavy metal remediation, as we could expect that with a careful choice of biochar lessened amounts would be needed to obtain a plant effect, resulting in fewer amounts of biochar added to the soil and consequently diminished pollutant immobilization. Another future line for this type of studies would be to use phytoremediators and biochars targeting at different heavy metals (or the biochar not to target any metal at all), as the biochar effect is strongly dependent on the chemical element immobilized.
